# Study of four Neotropical species of tree crickets *Oecanthus* Serville, 1831 (Orthoptera, Gryllidae) using cytogenetic and molecular markers

**DOI:** 10.1590/1678-4685-GMB-2021-0213

**Published:** 2022-04-29

**Authors:** Anelise Fernandes e Silva, Thays Duarte de Oliveira, Natasha Ávila Bertocchi, Vera Lúcia da Silva Valente, Edison Zefa, Maríndia Deprá

**Affiliations:** 1 Universidade Federal do Rio Grande do Sul, Instituto de Biociências, Departamento de Zoologia, Programa de Pós-Graduação em Biologia Animal, Porto Alegre, RS, Brazil.; 2 Universidade Federal do Rio Grande do Sul, Instituto de Biociências, Departamento de Genética, Programa de Pós-Graduação em Genética e Biologia Molecular, Porto Alegre, RS, Brazil.; 3 Universidade Federal de Pelotas, Instituto de Biologia, Programa de Pós-Graduação em Biologia Animal, Departamento de Zoologia, Ecologia e Genética, Capão do Leão, RS, Brazil.

**Keywords:** Insect, karyotype, Chromosome, Bayesian Inference, Oecanthinae

## Abstract

Karyotypes in the worldwide subfamily Oecanthinae show variations in diploid number, chromosome morphology, and sex-chromosome system. This study described the chromosome set and phylogenetic relationships of four Neotropical species, *Oecanthus lineolatus, O. valensis*, *O. pallidus*, and *O. pictus*. We used classical cytogenetics and Bayesian Inference for phylogenetic reconstruction, using the mitochondrial genes *COI*, *12S rRNA*, and *16S rRNA*; and analyzed the phylogenetic patterns of changes in chromosome numbers, using ChromEvol. We observed differences in chromosome number among species and two different sex-chromosome systems. *Oecanthus pictus* showed 2n = 21, X0♂/22, XX♀; *O. lineolatus*, 2n = 20, XY♂/XX♀; and *O. valensis* and *O. pallidus*, 2n = 18, XY♂/XX♀. The karyotype of *Oecanthus* was asymmetric, one group with large chromosomes and variation in heterochromatin distribution, and another with small acrocentric chromosomes. The phylogenetic tree recovered two main groups: one with the Palearctic species and another with species from different bioregions, but with low posterior probability. The Neotropical species grouped separately, *O. valensis* and *O. pictus* with Nearctic and Ethiopian species, and *O. pallidus* and *O. lineolatus* in another, well-supported clade. Together, the phylogenic and chromosome data suggest descending dysploidy events during the evolution of the group.

## Introduction

The order Orthoptera contains more than 28,000 described species, with a worldwide distribution. Species have been used as model organisms in several studies of cytogenetics, bioacoustics, and evolution (Hewitt, 1979; Bidau and Martí, 2010; Blackmon et al., 2017; Cigliano et al., 2021). Members of the suborder Ensifera show wide variation in chromosome number, ranging from 2n = 15 to 37 in Tettigoniidea (Warchałowska-Śliwa, 1998) and from 2n = 7 to 29 in Gryllidea (White, 1973; Hewitt, 1979; Mesa et al., 1982). Most of the species have the sex-chromosome system X0♂-XX♀, with the X chromosome usually showing positive heteropycnosis compared to the autosomes during prophase I of meiosis (White, 1978; Hewitt, 1979; Palacios-Gimenez et al., 2018). This differential heteropycnosis occurs through the earlier condensation of chromosome X in the initial phases of cell division (Bidau and Martí, 2010). X/autosome rearrangements may give rise to derived sex systems, from the original X0♂/XX♀ to Neo-XY♂/XX♀, X_1_X_2_Y♂-X_1_X_1_X_2_X_2_♀, and X_1_X_2_0♂-X_1_X_1_X_2_X_2_♀ (White, 1957; Saez, 1963; Mesa *et al.*, 2002; Zefa et al., 2014b; Palacios-Gimenez and Cabral-de-Mello, 2015).

Members of Oecanthinae are commonly known as “tree crickets”. *Oecanthus* Serville, 1831 is the largest genus, with 74 described species and a worldwide distribution (Walker, 1962; Cigliano et al., 2021). Described karyotypes of oecanthine crickets show variations in the chromosome number, morphology, and sex-chromosome system, although the chromosome sets of only eight species have been studied so far (Johnson, 1931; Makino, 1932; Ohmachi, 1935; Kitada, 1949; Hewitt, 1979; Milach et al., 2016; Zefa et al., 2018). One of these is *Neoxabea brevipes* Rehn, 1913, with 2n = 24 autosomes + two sex chromosomes (XY or X_1_X_2_0) (Zefa *et al.*, 2018); the other seven species belong to the genus *Oecanthus* (Johnson, 1931; Makino, 1932; Ohmachi, 1935; Kitada, 1949; Hewitt, 1979; Milach *et al.*, 2016). *Neoxabea brevipes* and *O. valensis* Milach and Zefa, 2016 inhabit the Neotropical bioregion; *O. nigricornis* Walker, 1869 and *O. quadripunctatus* Beutenmüller, 1894 the Nearctic; *O. longicauda* Matsumura, 1904 and *O. pellucens* (Scopoli, 1763) the Paleoarctic; and *O. indicus* Saussure, 1878 and *Oecanthus* sp. the Oriental (Aswanianarayana and Ashwath, 2005; Cigliano *et al.*, 2021).

The karyotypes for *O. indicus*, *O. nigricornis*, and *O. quadripunctatus* are 2n = 19, X0♂; for *O. longicauda,* and *O. pellucens*, 2n = 20, XY♂; and *Oecanthus* sp. and *O. valensis*, 2n = 18, XY♂. The species share an asymmetric karyotype that forms two groups of chromosomes according to size. The first group comprises large chromosomes, consisting of two or three autosome pairs and the X chromosome. The second comprises small (dot-like) chromosomes, including five, six, or seven autosome pairs and the Y chromosome, when the sex-chromosome system is XY (Johnson, 1931; Makino, 1932; Ohmachi, 1927, 1935; Kitada, 1949; Hewitt, 1979; Aswanianarayana and Ashwath, 2005; Milach et al., 2016).

Only Liu et al. (2018) previously dealt with the molecular evolution of the genus, reconstructing the phylogenetic relationships of the Cytochrome c Oxidase subunit I (COI) gene among species of *Oecanthus* from China. Using maximum-likelihood and Bayesian inference methods, Liu *et al.* (2018) found that the first separation occurred between *Oecanthus* ssp. and *Xabea levissima* Gorochov, 1992, both from the same subfamily. Within the genus, *O. antennalis* Liu, Yin and Xia, 1994 was the first to diverge and showed a close relationship to *O. longicauda* and *O. similator* Ichikawa, 2001; probably *O. similator* originated from the *O. longicauda* group (Liu *et al.*, 2018). In other phylogenetic studies, species of *Oecanthus* have been included in analyses to elucidate phylogenetic relationships in Ensifera, aiming to clarify the evolution of acoustic communication (Gwynne, 1995; Desutter-Grandcolas and Robillard, 2004; Jost and Shaw, 2006; Legendre et al., 2010; Song et al., 2015; Chintauan-Marquier et al., 2016).

The cytogenetics and phylogenetics of *Oecanthus* are little investigated, even though they show interesting chromosome variations and wide distributions, with species occurring in all bioregions. This study aimed to gain a more comprehensive insight into the evolutionary history of *Oecanthus*, describing the chromosome sets and phylogenetic relationships of *O. valensis*, *O. pallidus* Zefa, 2012, *O. lineolatus* Saussure, 1897, and *O. pictus* Milach and Zefa, 2015. We identified the chromosome number, sex-chromosome system, and heterochromatic regions using classical cytogenetic methods. Regarding molecular analysis, we used Bayesian Inference for phylogenetic reconstruction, using the mitochondrial genes. We then inferred phylogenetic relationships for the group and the pattern of changes in chromosome number during the course of evolution.

## Material and Methods

### Samples

Individuals of *O. valensis* were collected with a sweep net in shrubs and grasses, bordering highway BR101 alongside the conservation area “Reserva Natural Vale”, municipality of Linhares, state of Espírito Santo, Brazil on July 28, 2012 (Table 1). The specimens of *O. pictus*, *O. pallidus,* and *O. lineolatus* were collected in tobacco fields and on shrubs in the São João da Reserva district, municipality of São Lourenço do Sul, state of Rio Grande do Sul, Brazil in March 2012 (Table 1).

**Table 1-:** Specimen information and number of samples.

Species	Number and sex of individuals	Locality	Geographic coordinate
*O. valensis*	10♂ and 7♀	Linhares/ES	19º05’817’’S, 040º03’116’’W
*O. pictus*	20♂ and 5♀	São Lourenço do Sul/RS	31º17’39.43’’S, 52º09’02.76’’W
*O. lineolatus*	8♂ and 3♀	São Lourenço do Sul/RS	31º17’39.43’’S, 52º09’02.76’’W
*O. pallidus*	3♂ and 1♀	São Lourenço do Sul/RS	31º17’39.43’’S, 52º09’02.76’’W

ES, State of Espírito Santo, Brazil; RS, State of Rio Grande do Sul, Brazil

### Cytogenetic analyses

We obtained the chromosomes from testis follicles of males and from midguts of females and males, previously injected with 0.05% colchicine solution for 5 h, next in 0.075 KCl hypotonic solution for 5-10 min, and then fixed in Carnoy I (3 ethyl alcohol: 1 glacial acetic acid). We squashed the fixed material on the slide in 45% acetic acid and stained the chromosomes with 0.5% lacto-acetic orcein.

We used the C-banding technique of Sumner (1972). The slides were dipped into hydrochloric acid solution (0.1 N HCl) for 30 min at room temperature and rinsed with distilled water. Slides were then treated with 5% barium hydroxide at 60 °C for 3 min, washed in 0.2 N HCl for 2 min, and rinsed with distilled water. Next, slides were dipped in 2 x SSC solution at 60 °C for 45 min, washed with distilled water, and stained with 2% Giemsa in phosphate buffer (pH 6.8) for 10 min.

Meiosis and mitosis phases were selected and photographed with a Nikon S3200 digital camera mounted on an Olympus CX21 optical microscope. We calculated the centromere index according to Levan et al. (1964). For C-banding, slides were analyzed and photographed under a Zeiss Axiophot microscope using ZEN blue edition software. The generated map was constructed in the online platform SimpleMappr, figure edition, karyotype assembly, and the chromosome ideograms were constructed using the Adobe Photoshop CC 2015 program. 

### Molecular and phylogenetic analyses

DNA was extracted from the cricket hind femur and treated with a phenol/chloroform protocol, according to Jowett (1986). We amplified the genetic material using specific primers for Cytochrome c oxidase I (COI), 12S ribosomal RNA (rRNA), and 16S ribosomal RNA (rRNA), through the polymerase chain reaction (PCR). The primers used were HCO2198 and LCO1490 (Folmer et al., 1994), 12SF and 12SR (Kambhampati, 1995), and 16SAG and 16SBG (Robillard and Desutter-Grandcolas, 2006) for COI, 12S rRNA, and 16S rRNA, respectively (Table 2).

**Table 2 -:** Primers used for PCR amplification and sequencing, indicating the gene, described name, sequence, and source of each sequence primer.

Marker	Primers	Sequence	References
COI	LCO1490 HC02198	5’-GGTCAACAAATCATAAAGATATTGG-3’ 5’-TAAACTTCAGGGTGACCAAAAAATCA-3’	Folmer et al., 1994
12S rRNA	12SF 12SR	5’-TACTATGTTACGACTTAT-3’ 5’-AAACTAGGATTAGATACCC-3’	Kambhampati, 1995
16S rRNA	16SAG 16SBG	5’-CGCCTGTTTATCAAAAACATGT-3’ 5’-AGATCACGTAAGAATTTAATGGTC-3’	Robillard and Desutter-Grandcolas, 2006

The PCR assays were conducted with 50 ng of template DNA, 20 pM of each primer, 2.5 mM MgCl_2_, and 1 μL *Taq* DNA polymerase in a total volume of 50 μL. The reactions were amplified under the following conditions: first denaturation at 95 °C for 1 min, then 35 denaturation cycles at 95 °C for 1 min, 45 s for primer annealing at temperatures of 47-48 °C for COI, 44-45 °C for 12S, and 48-49 °C for 16S, then extension at 72 °C for 1 min, and a final extension at 72 °C for 5 min.

PCR products were visualized in 1% agarose gel and then purified with the EXO-SAP (UAB) enzymatic method for sequencing. The sequencing was performed both ways by the Sanger sequencing method at Macrogen Inc. (Seoul, South Korea). The chromatograms obtained were assembled and inspected using the Staden Package (Staden, 1996). We performed nucleotide BLAST, using a template for genes COI, 12S, and 16S in the National Center for Biotechnology Information (NCBI) (2022) to select *Oecanthus* sequences. We included in the phylogenetic analysis all the sequences available in GenBank for *Oecanthus* and for the outgroups, *Ceuthophilus* sp. Scudder, 1862 (Ensifera) and *Locusta migratoria* (Linnaeus, 1758) (Caelifera) (Table 3). We concatenated the sequences in head-to-tail sequence alignment, and for the species with unavailable genes, these were considered missing data. We used the software MEGA X 10.1 (Kumar et al., 2018) to align and edit the sequences.

**Table 3 -:** Species and accesses numbers of each sequence used in molecular analysis.

Species	COI	12S	16S	Bioregions
*O. antennalis* Liu, Yin and Xia, 1994	MH893702.1	-	-	Palearctic
*O. celerinictus* Walker, 1963	KM537641.1	-	-	Nearctic
*O. chopardi* Uvarov, 1957	-	-	KR903784.1	Ethiopian
*O. euryelytra* Ichikawa, 2001	MH893707.1	-	-	Palearctic
*O. exclamationis* Davis, 1907	MG436770.1	-	-	Nearctic
*O. fultoni* Walker, 1962	KR140441.1	-	-	Nearctic
O. lineolatus^*^ Saussure, 1897	MZ429066	MZ429327	MZ429331	Neotropical
*O. longicauda* Matsumura, 1904	MH893701.1	-	-	Palearctic
*O. nigricornis* Walker, 1869	KR143926.1	-	AF514469.1	Nearctic
*O. niveus* (De Geer, 1773)	KM535640.1	-	-	Nearctic
*O. oceanicus* He, 2018	MH893718.1	-	-	Palearctic
O. pallidus^*^ Zefa, 2012	-	MZ429328	MZ429332	Neotropical
*O. pellucens* (Scopoli, 1763)	HM422220.1	-	-	Palearctic
O. pictus^*^ Milach and Zefa, 2015	MZ429067	MZ429329	MZ429333	Neotropical
*O. pini* Beutenmüller, 1894	-	KJ024361.1	-	Nearctic
*O. quadripunctatus* Beutenmüller, 1894	MG466395.1	-	-	Nearctic
*O. rufescens* Serville, 1838	KX057720.1	KX057720.1	KX057720.1	Palearctic
*O. similator* Ichikawa, 2001	MH893700.1	-	-	Palearctic
*O. sinensis* Walker, 1869	NC_034799.1	NC_034799.1	NC_034799.1	Palearctic
*O. turanicus* Uvarov, 1912	MH893727.1	-	-	Palearctic
O. valensis^*^ Milach and Zefa, 2016	MZ429068	MZ429330	MZ429334	Neotropical
*O. zhengi* Xie, 2003	MH893715.1	-	-	Palearctic
*Ceuthophilus* sp. Scudder, 1862	HQ986388.1	KR903978.1	AF212056.1	-
*Locusta migratoria* (Linnaeus, 1758)	HQ986486.1	AB497582.1	JF932434.1	-

^*^Sequences generated in the present study.

(-) Missing data.

For the phylogenetic reconstructions, we used MrModeltest2 (Nylander, 2004) to determine the best-fit evolutionary model of substitution for each gene - the three partitions, according to the values of the Akaike information criterion (AIC). The best model for COI was GTR+I+G, for 12S rRNA was GTR+G, and for 16S rRNA was GTR+I+G. The analysis was run from 30 million generations, sampling every 30,000 generations, discarding the first 25% of the samples as burn-in. We performed the Bayesian Inference (BI) analysis in the program MrBayes 3.2.6 (Ronquist et al., 2011) on XSEDE in the online platform Cyberinfrastructure for Phylogenetic Research (CIPRES) (2021). In addition, to corroborate the findings in the BI, we performed a Neighbor-Joining analysis and phylogenetic reconstruction, employing each gene separately (data not shown).

### Chromosome number evolution

We used the software ChromEvol (Mayrose et al., 2010; Glick and Mayrose, 2014) to infer the chromosome evolution of *Oecanthus* along the phylogenetic tree recovered from the BI analysis. This software compares the fit of different models to biological data and may make it possible to gain insight into the pathways of chromosome-number evolution. For our data, the best evolution model determined by the program was DYS (dysploidy) according to the AIC values. The input files for analysis were the Bayesian phylogenetic tree, and the chromosome counts, with the name of each species and the haploid chromosome number (n). We included the *L. migratoria* outgroup chromosome information, with 2n = 23, X0 (Wei, 1958). We accepted two possible numbers for species with different haploid numbers for males and females, assuming a frequency of 0.5 for each one and that the proportion between males and females is the same. For taxa with an unknown chromosome number, we used the symbol “X” and considered this as missing data. Table 3 lists all species used in the molecular analysis and Table 4 lists the chromosome numbers. In Table 4, *O. indicus* and *Oecanthus* sp. were excluded from the ChromEvol analysis due to missing molecular data and no species specification, respectively.

**Table 4 -:** Available literature information: new results of karyotypes in *Oecanthus*, describing the diploid number, sex system, and morphologies for large chromosomes, the sexual X and Y.

Species	Morphologies of large chromosomes and chromosome Y					Diploid number	Sex System (♂/♀)	References
	1	2	3	X	Y			
O. indicus^**^	A	A	A	A	-	2n=19♂/20♀	X0/XX	Kitada, 1949; Nakamura and Kitada 1955
O. lineolatus^*^	M (40.9)	M (42.1)	-	SM (31.3)	Dot-like	2n=20♂/♀	XY/XX	Present study
*O. longicauda*	A	A	A	A	Dot-like	2n=20♂/♀	XY/XX	Hewitt, 1979; Ohmachi, 1927; Makino 1932
*O. nigricornis*	M	M	-	M	-	2n=19♂/20♀	X0/XX	Johnson, 1931
O. pallidus^*^	M (47.8)	M (47.4)	-	M (41.2)	ST (20)	2n=18♂/♀	XY/XX	Present study
*O. pellucens*	A	A	A	A	Dot-like	2n=20♂/♀	XY/XX	Hewitt, 1979
O. pictus^*^	M (45)	ST (16.66)	A (7.69)	M (45.45)	-	2n=21♂/22♀	X0/XX	Present study
*O. quadripunctatus*	M	M	-	M	-	2n=19♂/20♀	X0/XX	Beaudry, 1973
*Oecanthus* sp.^**^	A	A	A	M	Dot-like	2n=18♂/♀	XY/XX	Aswanianarayana and Ashwath, 2005
O. valensis^*^	M (48)	SM (36.8)	-	SM (25.9)	Dot-like	2n=18♂/♀	XY/XX	Milach et al., 2016; Present study

^*^Centromeric indexes were calculated for the species of the present study.

M - Metacentric/ SM - Submetacentric/ ST - Subtelocentric/ A - Acrocentric.

^**^Not included in ChromEvol analysis.

## Results

### Karyotyping and C-banding

**Figure 1 -:**
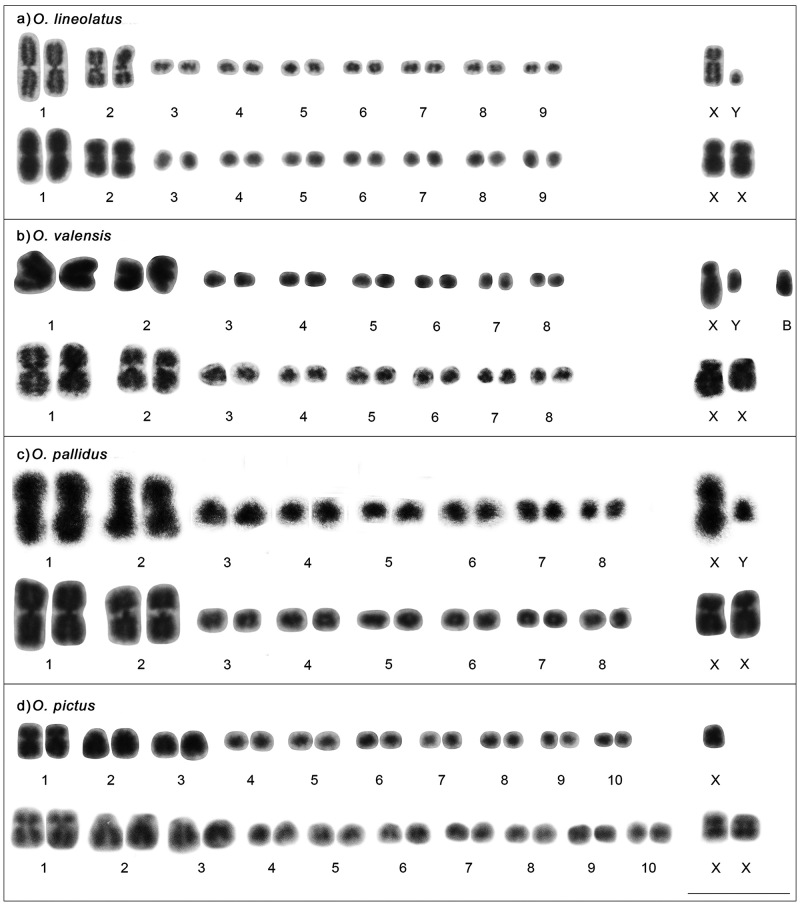
Mitotic karyotype of (a) *Oecanthus lineolatus* with 2n = 20, XY♂/ XX♀; (b) *O. valensis* with 2n = 18, XY♂/ XX♀ and one chromosome B in the male karyotype; (c) *O. pallidus* with 2n = 18, XY♂/ XX♀; and (d) *O. pictus* with 2n = 21, X0♂/ 22, XX♀. Scale bar = 10 µm.

**Figure 2 -:**
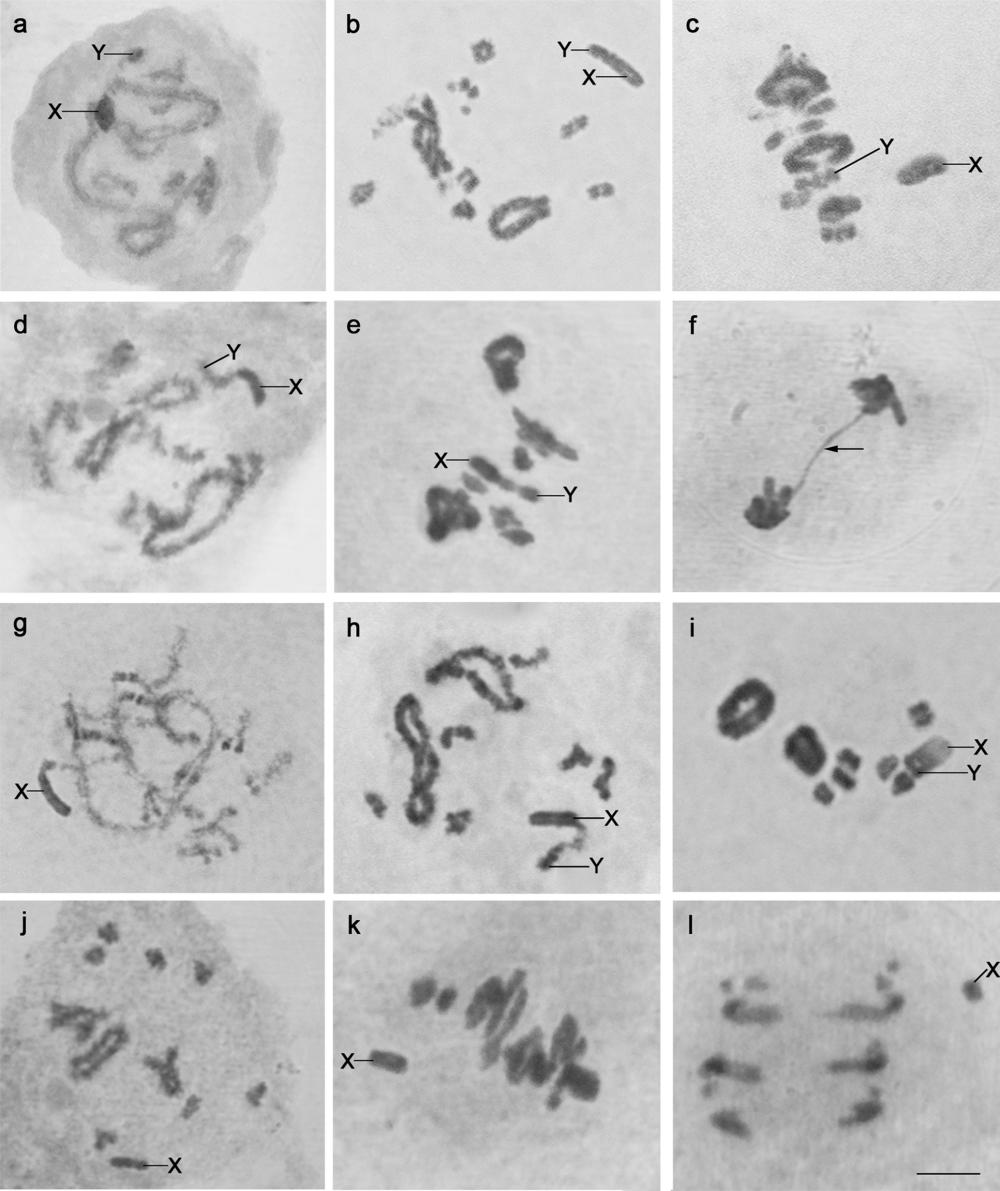
Meiotic phases of male individuals indicating the behavior of the sex chromosomes: a-c) *Oecanthus lineolatus* during (a) Pachytene, (b) Diplotene, and (c) Metaphase I; d-f) *O. valensis* during (d) Diplotene, (e) Metaphase I, and (f) Anaphase II (arrow indicates the chromatin bridge observed); g-i) *O. pallidus* during (g) Pachytene, (h) Diplotene, and (i) Metaphase I; and j-l) *O. pictus* during (j) Diplotene, (k) Metaphase I, and (l) Anaphase I. Scale bar = 10 µm.

*Oecanthus lineolatus* showed a diploid number of 2n = 20, XY♂/XX♀, with two pairs of large metacentric autosomes (Table 4), pair 2 with a secondary constriction in the interstitial region, and seven pairs of small chromosomes (Figure 1a). The X chromosome was large and submetacentric (Table 4), and the Y chromosome was one of the smallest (Figure 1a). During meiosis I, the sex chromosomes behaved as bivalents, forming chiasma in prophase I (Figure 2a, b), positioning together in the equatorial plate in metaphase I, and each migrating to opposite poles of the cell in anaphase I (Figure 2c). In pachytene, the sex chromosomes were heterochromatic at the ends and with a euchromatic region between them. In diplotene, the chromosome of pair 2 showed elastic constrictions, which may correspond to secondary constrictions (Figure 2b).

*Oecanthus valensis* had 2n = 18, XY♂/XX♀, with two pairs of large meta/submetacentric autosomes and six pairs of small chromosomes (Figure 1b and Table 4). The sex-chromosome system had a large submetacentric X (Table 4) and a small Y chromosome (Figure 1b), both attached by a terminal chiasma during prophase I. The X was more heteropycnotic than the Y, and both showed a gradual increase in heterochromatinization during prophase I (Figure 2d, e). Some cells of one individual exhibited a B chromosome (Figure 1b), and in another individual the cells formed a chromatin bridge during anaphase/telophase II (Figure 2f).

*Oecanthus pallidus* had 2n = 18, XY♂/XX♀, with two pairs of large metacentric chromosomes (Table 4) and six small autosomal pairs (Figure 1c). The X chromosome was large and metacentric, and the Y was small and subtelocentric (Figure 1c and Table 4). We observed the morphology of chromosome Y only in *O. pallidus* because it was larger and it was possible to locate the centromere position. In contrast, the Y was not well defined in the other species, showing a dot-like morphology. In pachytene, chromosome X was heterochromatic and Y was euchromatic, and they appeared not to be paired. In diplotene I, the sex chromosomes behaved as heteromorphic bivalents and were heteropycnotic positive, with a euchromatic segment between them (Figure 2g). There was a gradual increase in heterochromatinization of X and Y segments in prophase I (Figure 2g, h), and in metaphase I they moved together on the equatorial plate (Figure 2i).

In specimens of *O. pictus*, the diploid number was 2n = 21, X0♂, and 2n = 22, XX♀, with three pairs of large autosomes, one metacentric, one subtelocentric, and one acrocentric; and seven pairs of small autosomes (Figure 1d and Table 4). The X chromosome was large and metacentric (Table 4), behaving as univalent during cell division (Figure 2j, k, l) and migrating to one of the cell poles in anaphase I (Figure 2l). In diplotene I, the sex chromosome showed positive heteropycnosis in comparison with the autosomes (Figure 2j).

**Figure 3-:**
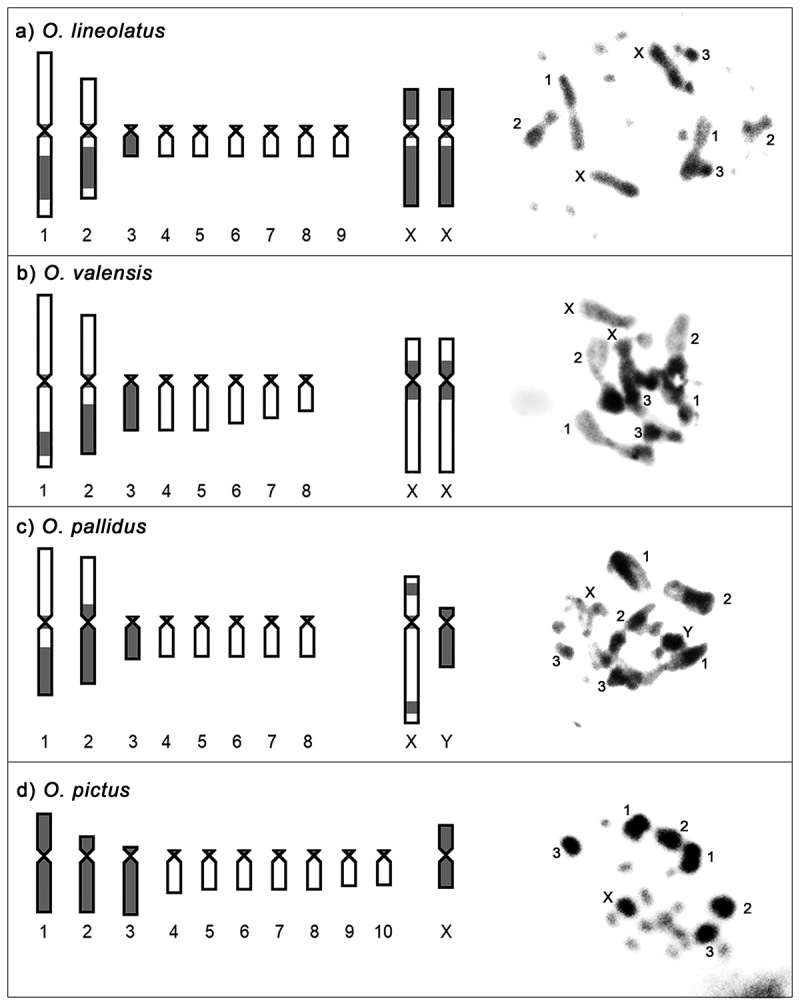
Identification of C-banding markers (gray) in mitotic metaphase of females of (a) *Oecanthus lineolatus* and (b) *O. valensis*, and males of (c) *O. pallidus* and (d) *O. pictus*. Indication of chromosome pairs with C-banding markers. Scale bar = 10 µm.

The C-banding pattern showed that in all species, the small chromosomes were acrocentric with a small pericentromeric C-band at one end, except for pair 3 in *O. lineolatus*, *O. valensis*, and *O. pallidus* that showed a heterochromatic block (Figure 3a, b, c). The chromosomes X had a high degree of heterochromatinization differing between the species (Figure 3). Variations were observed in the large autosomes, such as in *O. lineolatus,* where pair 1 had an interstitial band and pair 2 exhibited a heterochromatic block in a secondary constriction (Figure 3a). *Oecanthus valensis* had an interstitial band in the bivalents of pair 1 and a heterochromatic block in pair 2 (Figure 3b). *Oecanthus pallidus* had a C-band in the telomeric region of pair 1 and a pericentric heterochromatin block in pair 2, and the Y chromosome was heterochromatic (Figure 3c). C-banding in *O. pictus* showed high heterochromatinization of the three large chromosomes (Figure 3d).

### Phylogenetic reconstructions

The consensus tree (obtained from COI, 12S rRNA, and 16S rRNA concatenated mitochondrial fragments) enabled us to infer the phylogenetic relationships of *O. lineolatus*, *O. pallidus*, *O. valensis,* and *O. pictus* with the other species. The phylogenetic tree showed two main groups: one composed only of species from the Palearctic bioregion (except *O. antennalis*), and the other group composed of the remaining species from different bioregions, although this separation was not strongly supported (p.p. value = 0.55). In the Palearctic group, high posterior probabilities and close relationships were recovered mainly between *O. similator* and *O. longicauda*, and among *O. euryelytra* Ichikawa, 2001, *O. sinensis* Walker, 1869*,* and *O. rufescens* Serville, 1838.

**Figure 4-:**
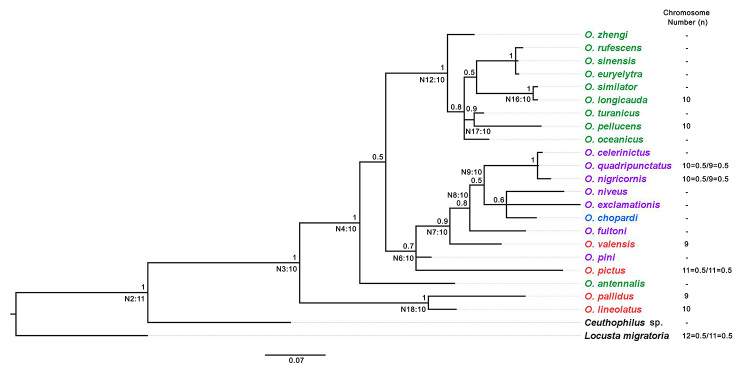
Bayesian Inference using mitochondrial concatenated data (COI, 12S rDNA, and 16S rDNA) in *Oecanthus* species. Colors indicate each bioregion: green, species from the Palearctic bioregion; purple, Nearctic; red, Neotropical; and blue, Ethiopian. The outgroups were *Ceuthophilus* sp. and *Locusta migratoria*. Above each branch are indicated the posterior probabilities; (N) represents the node names and the ancestral haploid chromosome number inferred by ChromEvol software. Chromosome haploid number of living species, and (-) represents missing data for karyotype.

Regarding the Neotropical species, *O. pallidus* and *O. lineolatus* showed a close, strongly supported relationship (p.p. value = 1.00), and *O. valensis* and *O. pictus* grouped with species from the Nearctic and Ethiopian regions. In this group, only *O. quadripunctatus*, *O. nigricornis*, and *O. celerinictus* Walker, 1963 from the Nearctic region were closely related and showed high posterior probability (p.p. value = 1.00) (Figure 4).

### Chromosome evolution along the phylogenetic tree

Concerning karyotype evolution, we used ChromEvol and based the analysis on chromosome number and molecular markers. Chromosome data (Table 4) indicated that the transition occurring in the genus is descending dysploidy, indicating a process of chromosome loss along the tree. There were four main loss events with significances greater than 0.5, in the ancestral nodes N2 (0.51) and N3 (0.58) and in the species *O. valensis* (0.65) and *O. pallidus* (1.00) (Figure 4). The program inferred that the ancestral node N2 may have an n = 11 and for the ancestral N3 was then reduced, to n = 10. The ancestral nodes along the branches maintained the haploid number of n = 10, until a significant loss in *O. valensis* and *O. pallidus*, both with n = 9 (Figure 4).

## Discussion

**Figure 5 -:**
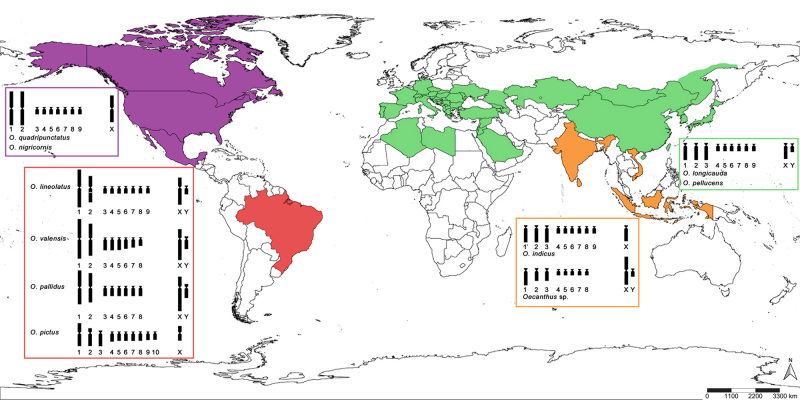
Map showing the distribution of *Oecanthus* species with karyotypes analyzed. In green, *O. longicauda* and *O. pellucens* from the Palearctic bioregion, both with 2n = 20, XY♂ (Hewitt 1979; Ohmachi 1927; Makino 1932). In purple, *O. nigricornis* and *O. quadripunctatus* from the Nearctic bioregion, both with 2n = 19, X0♂ (Johnson 1931; Beaudry 1973). In orange, species from the Oriental bioregion, *O. indicus* with 2n = 19, X0♂, and *Oecanthus* sp. with 2n = 18, XY♂ (Kitada 1949; Nakamura and Kitada 1955; Aswanianarayana and Ashwath 2005). In red, species from the Neotropical bioregion, *O. lineolatus* with 2n = 20, XY♂; *O. valensis* and *O. pallidus* with 2n = 18, XY♂; and *O. pictus* with 2n = 21, X0♂.

Cytogenetic studies with *Oecanthus* comprise species from four bioregions, Palearctic, Nearctic, Neotropical, and Oriental (Figure 5) (Aswanianarayana and Ashwath, 2005; Cigliano et al., 2021). We described for the first time the karyotypes of *O. pallidus*, *O. lineolatus*, and *O. pictus*, all from the Neotropical region (Figure 1). We found that the sex-chromosome system and diploid number were the same for *O. valensis* and *O. pallidus*, with 2n = 18, XY♂; *O. lineolatus* had the same system, with a different diploid number, 2n = 20, XY♂; and *O. pictus* had the most distinct karyotype, with 2n = 21, X0♂ (Figure 1).

We found that *O. pallidus* had the smallest diploid number, 2n = 18, as previously reported for *O. valensis* and *Oecanthus* sp., and *O. pictus* had the highest chromosome number in the genus, with 2n = 21. The other species did not show wide chromosome variability, with diploid numbers ranging from 2n = 19 to 20 (Johnson, 1931; Makino, 1932; Ohmachi, 1927, 1935; Kitada, 1949; Hewitt, 1979; Aswanianarayana and Ashwath, 2005; Milach et al., 2016). The sex-chromosome system was as previously described for the other species of the genus (X0 and XY) (Johnson, 1931; Makino, 1932; Ohmachi, 1927, 1935; Kitada, 1949; Hewitt, 1979; Aswanianarayana and Ashwath, 2005; Milach *et al.*, 2016).

The Neotropical species showed two pairs of large metacentric chromosomes, similar to the Nearctic species and different from the Oriental and Palearctic species with large acrocentric chromosomes (Figure 5 and Table 4) (Johnson, 1931; Makino, 1932; Ohmachi, 1927, 1935; Kitada, 1949; Nakamura and Kitada, 1955; Montalenti et al., 1965; Beaudry, 1973; Hewitt, 1979). The large chromosomes in *O. pictus* differed from other karyotypes, with one metacentric, one submetacentric, and one acrocentric pair (Figure 5 and Table 4). Our results for *O. valensis* were congruent with those of Milach et al. (2016) (Table 4). B chromosomes were observed in only two species of the genus; in *O. valensis* they were small and larger than chromosome Y (Figure 1b), and in *O. pellucens* were small and similar in size to chromosome Y (Hewitt, 1979; Milach *et al.*, 2016).

Both kinds of sex-chromosome systems, X0 and XY, occurred in the Neotropical species. This variation was also seen in the species from the Oriental Region, while the Nearctic species possess only the X0 mechanism, and in the Palearctic only the XY (Figure 5) (Johnson, 1931; Makino, 1932; Ohmachi, 1927, 1935; Kitada, 1949; Nakamura and Kitada, 1955; Beaudry, 1973; Hewitt, 1979; Aswanianarayana and Ashwath, 2005, Milach et al., 2016).

Evolutionarily, it is expected that fusions will occur between chromosomes, reducing the diploid number and forming bi-armed chromosomes (metacentric or submetacentric). In chromosome changes, fusion processes are expected to be more common than fissions (Baker and Bickham, 1980; Hemp et al., 2013). Considering this and the analysis of chromosome evolution along the phylogenetic tree, the chromosome set of *O. valensis* and *O. pallidus* appears to be the most derived, with the smallest diploid number in the group and an XY sex-chromosome system. Although *Oecanthus* sp. (Oriental) shows the same diploid number and sex-chromosome system as both Neotropical species, the chromosome morphology set (acrocentric) indicated a less-derived condition (Aswanianarayana and Ashwath, 2005). 

The XY sex-chromosome system of *O. valensis*, *O. pallidus*, and *O. lineolatus* probably derived from a centric fusion rearrangement between a large X-acrocentric chromosome with a small bivalent pair (White, 1954, 1957; Saez, 1963; Rice, 1996; Kaiser and Bachtrog, 2010; Castillo et al., 2010; Palacios-Gimenez et al., 2015b, 2018). The X chromosome and the autosomes undergo breaks and fusion, forming a metacentric and a small chromosome; the latter is composed of centromere regions and is usually lost during cell divisions. Chromosome X becomes a bi-armed chromosome, formed by fusion of the acrocentric X and the autosome, and the free autosome starts to behave similarly to the Y chromosome (Saez, 1963; Hewitt, 1979). During the meiotic prophase, the Y chromosome will pair with its homologue, which fused with the X chromosome, as occurs during pachytene and diplotene of the grasshopper *Ronderosia bergii* (Stål, 1878) (Palacios-Gimenez *et al.*, 2015b). In contrast, *O. pictus* has the X0 mechanism, and the X is metacentric and smaller than in the other three species.

In the XY sex-chromosome system of the Neotropical species of *Oecanthus,* a euchromatic segment occurs between two heterochromatic segments in the initial phases of meiosis. The euchromatic part is referent to the chiasma between the Y chromosome and its homologue fused with the X chromosome. The X/autosome rearrangement accompanied a gradual loss of crossing over between autosomal homologues and gradual heterochromatinization of the autosomal arm on the X chromosome (Saez, 1963). This process of heterochromatinization is typical in the evolution of sex chromosomes and indicates that the greater the degree of heterochromatinization in the segments of the XY mechanism, the older the origin of the rearrangement (White, 1951; Saez, 1963; Rice, 1996; Mesa et al., 2001).

Using the C-banding technique for the first time in chromosomes of *Oecanthus*, we found different patterns in the large chromosomes among species (Figure 3). For *O. lineolatus*, we observed a large heterochromatic block in the secondary constriction of pair 2, as also seen for the karyotypes of *Gryllus assimilis* (Fabricius, 1775) and *Eneoptera surinamensis* (De Geer, 1773) (Palacios-Gimenez et al., 2015a) (Figure 3a). *Oecanthus lineolatus*, *O. valensis*, and *O. pallidus* showed heterochromatic bands for pairs 1 and 2. Pair 1 in *O. lineolatus* and *O. valensis* was in the interstitial region, and in *O. pallidus* was in the telomere (Figure 3b, c). The telomere bands also differed from the findings for the cricket *G. assimilis* and the grasshopper *Paracinipe* sp. Descamps and Maunassif, 1972, where they occurred only in medium and small chromosomes (Palacios-Gimenez *et al.*, 2015a; Buleu et al., 2019). The Y chromosome in *O. pallidus* is entirely heterochromatic, and the Neo-Y of *R. bergii* shows the same pattern (Palacios-Gimenez *et al.*, 2015b). This pattern may be related to repeated DNA accumulation in this chromosome, changing the heterochromatin structure (Figure 3c) (Palacios-Gimenez *et al.*, 2015b). In the bushcricket *E. surinamensis*, the heterochromatin showed a different pattern, occurring as dispersed blocks in the Neo-Y (Ferreira and Cella 2006; Palacios-Gimenez *et al*., 2015a).

The chromatin bridge in anaphase II of *O. valensis* occurs in other species of Orthoptera, usually related to chromosome breaks and rearrangements (Figure 2f) (Warchałowska-Śliwa et al., 2005; Zefa et al., 2014a). Chromatin bridges are chromatin segments positioned parallel to the segregating chromosomes during anaphase II (Fenech et al., 2011, Bizard and Hickson, 2018). Usually, they form due to dicentric chromatin manifestations, where each centromere is segregating to an opposite pole of the cell (Acilan et al., 2007; Bizard and Hickson, 2018). Chromatin bridges may cause cell instability, lead to cell death, and be related to fecundity reduction (Kirkpatrick and Barton, 2006; Bizard and Hickson, 2018). Also, when the bridge breaks, it usually generates daughter cells with unbalanced copies of genes due to uneven breaking of the chromatin segment and rearrangements between chromosomes such as translocations and deletions (Acilan *et al.*, 2007; Fenech *et al.*, 2011).

Using molecular analysis, this study is the first to recover the phylogenetic relationships of *Oecanthus* from different bioregions. According to the BI, *O. longicauda* and *O. similator* are phylogenetically close, and probably *O. similator* originated from a group of *O. longicauda* (Liu et al., 2018). As previously found by Liu *et al*. (2018), *O. antennalis* was positioned separately from other species from the Palearctic region. The Neotropical species *O. pallidus* and *O. lineolatus* shared the same distribution and showed a close phylogenetic relationship. *Oecanthus pictus*, also from southern Brazil, appeared to be little related to these species. *Oecanthus valensis*, from southeastern Brazil, was more closely associated with the Nearctic than the Neotropical species.

Species of *Oecanthus* have an uncertain phylogenetic position within Oecanthinae; they appear close to the *Neoxabea-Xabea* group due to their general form, which may be related to adaptive issues. Therefore, the morphologic pattern found in this genus could be highly conserved, independently of their distribution (Desutter-Grandcolas, 1990). Such as the pigmented spots on the legs of *O. valensis* that are observed in only a few species, among them *O. niveus*, *O. celerinictus*, and *O. bakeri* Collins et al. (2014) (Walker, 1963; Collins *et al.*, 2014; Milach et al., 2016). All these species occur in the Nearctic and Neotropical bioregions, and *O. valensis* grouped in the same clade as *O. niveus* and *O. celerinictus* (Cigliano et al., 2021).

The analysis to identify patterns of change in chromosome number in the course of evolution showed four loss events, with high significance, indicating a reduction in the chromosome number. These events were highly important for the reduced diploid number found in *O. valensis* and *O. pallidus*. The decrease may be due to rearrangements and fusion processes between chromosomes (Baker and Bickham, 1980; Hemp et al., 2013). Similar processes occurred in other orthopteran species, as in the genus *Dichroplus* Stål, 1873, where the accumulation of fusions between autosome-autosome and X-autosome led to modifications of the ancestral chromosome set of 2n = 22 + X0♂/XX♀ to the reduced karyotypes of *D. pratensis* Bruner, 1900 (2n = 20) and *D. obscurus* Bruner, 1900 (2n = 18) (Colombo et al., 2005).

The present study is the first to describe the karyotypes of *O. pallidus*, *O. lineolatus*, and *O. pictus*, and also to use banding techniques in karyotypes of *Oecanthus* and analyze the relationship of this group using individuals from different bioregions. We found variations in the diploid number and two sex-chromosome systems in the genus. Among the species, *O. pictus* shows distinct chromosome characteristics in the diploid number and morphology. Two of the species that have been studied are Palearctic, two Nearctic, one Neotropical, and two Oriental. The molecular and cytogenetic data indicated that the process of descending dysploidy is the most probable event for chromosome evolution along the phylogenetic tree. Future cytogenetic and molecular studies involving more species of *Oecanthus* are needed to comprehend the chromosome and group evolution.

## References

[B1] Acilan C, Potter DM, Saunders WS (2007). DNA repair pathways involved in anaphase bridge formation. Genes Chromosomes Cancer.

[B2] Aswanianarayana NV, Ashwath S (2005). Karyotype characteristics of forty-one species of Orthoptera and their evolutionary trends at the family level. Rec Zool Surv India.

[B3] Baker RJ, Bickham JW (1980). Karyotypic evolution in bats: evidence of extensive and conservative chromosomal evolution in closely related taxa. Syst Biol.

[B4] Beaudry JR (1973). Une analyse des complements chromosomiques de certains orthopteres du Quebec et sa signification taxonomique et evolutionnaire. Can J Genet Cytol.

[B5] Bidau CJ, Martí DA (2010). 110 years of orthopteran cytogenetics, the chromosomal evolutionary viewpoint, and Michael White’s signal contributions to the field. J Orthoptera Res.

[B6] Bizard AH, Hickson ID (2018). Anaphase: A fortune-teller of genomic instability. Curr Opin Cell Biol.

[B7] Blackmon H, Ross L, Bachtrog D (2017). Sex determination, sex chromosomes, and karyotype evolution in insects. J Hered.

[B8] Buleu OG, Jetybayev IY, Chobanov DP, Bugrov AG (2019). Comparative analysis of C-heterochromatin, ribosomal and telomeric DNA markers in chromosomes of Pamphagidae grasshoppers from Morocco. Comp Cytogenet.

[B9] Castillo ER, Marti DA, Bidau CJ (2010). Sex and neo-sex chromosomes in Orthoptera: A review. J Orthoptera Res.

[B10] Chintauan‐Marquier IC, Legendre F, Hugel S, Robillard T, Grandcolas P, Nel A, Zuccon D, Desutter‐Grandcolas L (2016). Laying the foundations of evolutionary and systematic studies in crickets (Insecta, Orthoptera): A multilocus phylogenetic analysis. Cladistics.

[B11] Cigliano MM, Braun H, Eades DC, Otte D (2021). Orthoptera Species File Online.

[B12] Collins N, van den Berghe E, Carson L (2014). Two new species of Neoxabea, three new species of Oecanthus, and documentation of two other species in Nicaragua (Orthoptera: Gryllidae: Oecanthinae). T Am Entomol Soc.

[B13] Colombo P, Cigliano MM, Sequeira AS, Lange CE, Vilardi JC, Confalonieri VA (2005). Phylogenetic relationships in Dichroplus Stål (Orthoptera: Acrididae: Melanoplinae) inferred from molecular and morphological data: testing karyotype diversification. Cladistics.

[B14] Desutter-Grandcolas L (1990). Etude phylogénétique biogéographique et écologique des Grylloidea néotropicaux (Insectes Orthoptères)..

[B15] Desutter-Grandcolas L, Robillard T (2004). Acoustic evolution in crickets: need for phylogenetic study and a reappraisal of signal effectiveness. An Acad Bras Cienc.

[B16] Fenech M, Kirsch-Volders M, Natarajan AT, Surralles J, Crott JW, Parry J, Norppa H, Eastmond DA, Tucker JD, Thomas P (2011). Molecular mechanisms of micronucleus, nucleoplasmic bridge and nuclear bud formation in mammalian and human cells. Mutagenesis.

[B17] Ferreira A, Cella DM (2006). Chromosome structure of Eneoptera surinamensis (Orthoptera, Grylloidea, Eneopterinae) as revealed by C, NOR and N banding techniques. Chromosome Sci.

[B18] Folmer O, Black M, Hoeh W, Lutz R, Vrijenhoek R (1994). DNA primers for amplification of mitochondrial cytochrome c oxidase subunit I from diverse metazoan invertebrates. Mol Mar Biol Biotechnol.

[B19] Glick L, Mayrose I (2014). ChromEvol: Assessing the pattern of chromosome number evolution and the inference of polyploidy along a phylogeny. Mol Biol Evol.

[B20] Gwynne DT (1995). Phylogeny of the Ensifera (Orthoptera): A hypothesis supporting multiple origins of acoustical signalling, complex spermatophores and maternal care in crickets, katydids, and weta. J Orthoptera Res.

[B21] Hemp C, Heller KG, Warchałowska-Śliwa E, Hemp A (2013). The genus Aerotegmina (Orthoptera, Tettigoniidae, Hexacentrinae): Chromosomes, morphological relations, phylogeographical patterns and description of a new species. Org Divers Evol.

[B22] Hewitt GM, Jolui B (1979). Animal Cytogenetics 3. Insecta I.

[B23] Johnson HH (1931). Centrioles and other cytoplasmic components of the male germ cells of the Gryllidae. Z Wiss Zool.

[B24] Jost MC, Shaw KL (2006). Phylogeny of Ensifera (Hexapoda: Orthoptera) using three ribosomal loci, with implications for the evolution of acoustic communication. Mol Phylogenet Evol.

[B25] Jowett T, Roberts DB (1986). Drosophila: A practical approach.

[B26] Kaiser VB, Bachtrog D (2010). Evolution of sex chromosomes in insects. Annu Rev Genet.

[B27] Kambhampati S (1995). A phylogeny of cockroaches and related insects based on DNA sequence of mitochondrial ribosomal RNA genes. Proc Natl Acad Sci.

[B28] Kirkpatrick M, Barton N (2006). Chromosome inversions, local adaptation and speciation. Genetics.

[B29] Kitada S (1949). Preliminary notes on the chromosomes of Oecanthus indicus. Kromosomo.

[B30] Kumar S, Stecher G, Li M, Knyaz C, Tamura K (2018). MEGA X: Molecular evolutionary genetics analysis across computing platforms.. Mol Biol Evol.

[B31] Legendre F, Robillard T, Song H, Whiting MF, Desutter‐Grandcolas L (2010). One hundred years of instability in ensiferan relationships.. Syst Entomol.

[B32] Levan A, Fredga K, Sandberg AA (1964). Nomenclature for centromeric position on chromosomes. Hereditas.

[B33] Liu XT, Jing J, Xu Y, Liu YF, He ZQ (2018). Revision of the tree crickets of China (Orthoptera: Gryllidae: Oecanthinae). Zootaxa.

[B34] Makino S (1932). An unequal pair of idiochromosomes in the tree-cricket, Oecanthus longicauda Mats. J Fac Sci Hokkaido Univ Ser VI Zool.

[B35] Mayrose I, Barker MS, Otto SP (2010). Probabilistic models of chromosome number evolution and the inference of polyploidy. Syst Biol.

[B36] Mesa A, Ferreira A, Carbonell CS (1982). Cariologia de los Acridiodeos neotropicales: estado actual de su conocimiento y nuevas contribuciones. Ann Soc Entomol Fr.

[B37] Mesa A, Fontanetti CS, García-Novo P (2001). Does an x-autosome centric fusion in Acridoidea condemn the species to extinction?. J Orthoptera Res.

[B38] Mesa A, García-Novo P, Dos Santos D (2002). X1X2O (male)-X1X1X2X2 (female) chromosomal sex-determining mechanism in the cricket Cicloptyloides americanus (Orthoptera, Grylloidea, Mogoplistidae).. J Orthoptera Res.

[B39] Milach EM, Costa MKM, Martins LP, Nunes LA, Silva DSM, Garcia FRM, De Oliveira EC, Zefa E (2016). New species of tree cricket Oecanthus Serville, 1831 (Orthoptera: Gryllidae: Oecanthinae) from Reserva Natural Vale, Espírito Santo, Brazil, with chromosome complement. Zootaxa.

[B40] Montalenti G, Rocchi A, Fontana PG (1965). IL Corredo cromosomico di Oecanthus pellucens (ORTHOPTERA GRYLLOIDEA). Rend Lincei-Mat Appl.

[B41] Nakamura K, Kitada JI (1955). Chromosomes of some orthopteroid insects, with special reference to sex-chromosomes. Cytologia.

[B42] Nylander JAA (2004). Program distributed by the author. Evolutionary Biology Centre.

[B43] Ohmachi F (1927). Preliminary note on cytological studies on Grylloidea. Proc Imp Acad.

[B44] Ohmachi F (1935). A comparative study of chromosome complements in the Grylloidea in relation to taxonomy. Bull Mie Imp Coll Agric For.

[B45] Palacios-Gimenez OM, Cabral-de-Mello DC (2015). Repetitive DNA chromosomal organization in the cricket Cycloptiloides americanus: A case of the unusual X1X20 sex chromosome system in Orthoptera. Mol Genet Genomics.

[B46] Palacios-Gimenez OM, Carvalho CR, Soares FAF, Cabral-de-Mello DC (2015). Contrasting the chromosomal organization of repetitive DNAs in two Gryllidae crickets with highly divergent karyotypes. PLoS One.

[B47] Palacios-Gimenez OM, Marti DA, Cabral-de-Mello DC (2015). Neo-sex chromosomes of Ronderosia bergi: Insight into the evolution of sex chromosomes in grasshoppers. Chromosoma.

[B48] Palacios-Gimenez OM, Milani D, Lemos B, Castillo ER, Martí DA, Ramos E, Martins C, Cabral-de-Mello DC (2018). Uncovering the evolutionary history of neo-XY sex chromosomes in the grasshopper Ronderosia bergii (Orthoptera, Melanoplinae) through satellite DNA analysis. BMC Evol Biol.

[B49] Rice WR (1996). Evolution of the Y sex chromosome in animals. Bioscience.

[B50] Robillard T, Desutter-Grandcolas L (2006). Phylogeny of the cricket subfamily Eneopterinae (Orthoptera, Grylloidea, Eneopteridae) based on four molecular loci and morphology. Mol Phylogenet Evol.

[B51] Ronquist F, Huelsenbeck J, Teslenko M (2011). Draft MrBayes version 3.2 manual: Tutorials and model summaries.

[B52] Saez FA (1963). Gradient of heterochromatinization in the evolution of the sexual system “neo-X neo-Y”. Port Acta Biol Ser A.

[B53] Song H, Amédégnato C, Cigliano MM, Desutter‐Grandcolas L, Heads SW, Huang Y, Otte D, Whiting MF (2015). 300 million years of diversification: elucidating the patterns of orthopteran evolution based on comprehensive taxon and gene sampling. Cladistics.

[B54] Staden R (1996). The Staden sequence analysis package. Mol Biotechnol.

[B55] Sumner AT (1972). A simple technique for demonstrating centromeric heterochromatin. Exp Cell Res.

[B56] Walker TJ (1962). The taxonomy and calling songs of United States tree crickets (Orthoptera: Gryllidae: Oecanthinae). I. The genus Neoxabea and the niveus and varicornis groups of the genus Oecanthus. Ann Entomol Soc Am.

[B57] Walker TJ (1963). The taxonomy and calling songs of United States tree crickets (Orthoptera: Gryllidae: Oecanthinae). II. The nigricornis group of the genus Oecanthus. Ann Entomol Soc Am.

[B58] Warchałowska-Śliwa E (1998). Karyotype characteristics of katydid orthopterans (Ensifera, Tettigoniidae) and remarks on their evolution at different taxonomic levels. Folia Biol.

[B59] Warchałowska-Śliwa E, Niklińska M, Görlich A, Michailova P, Pyza E (2005). Heavy metal accumulation, heat shock protein expression and cytogenetic changes in Tetrix tenuicornis (L.) (Tetrigidae, Orthoptera) from polluted areas. Environ Pollut.

[B60] Wei H (1958). Cytological studies on migratory locust hybrid, Locusta migratoria migratoria L. Locusta migratoria manilensis Meyen. Acta Zool Sinica.

[B61] White MJD (1951). Cytogenetics of orthopteroid insects. Adv Genet.

[B62] White MJD (1954). Animal cytology and evolution.

[B63] White MJD (1957). Cytogenetics and systematic entomology. Annu Rev Entomol.

[B64] White MD (1973). Animal cytology and evolution.

[B65] White MJD (1978). Chain processes in chromosomal speciation. Syst Zool.

[B66] Zefa E, Acosta RC, Timm VF, Szinwelski N, Marinho MAT, Da Costa MKM (2018). The Tree Cricket Neoxabea brevipes Rehn, 1913 (Orthoptera: Gryllidae: Oecanthinae) from the Brazilian southern Atlantic Forest: Morphology, bioacoustics, and cytogenetics. Zootaxa.

[B67] Zefa E, Cordeiro J, Blauth M, Piumbini M, Silva AF, Costa MKM, Martins LDP (2014). Expanding the geographic cytogenetic studies in the bush crickets Eneoptera surinamensis (De Geer, 1773) (Orthoptera, Gryllidae, Eneopterinae) from Brazilian Atlantic and Amazon Forest. Zootaxa.

[B68] Zefa E, Redu DR, Costa MKM, Fontanetti CS, Gottschalk MS, Padilha GB, Silva AF, Martins LP (2014). A new species of Endecous Saussure, 1878 (Orthoptera, Gryllidae) from northeast Brazil with the first X1X20 chromosomal sex system in Gryllidae. Zootaxa.

[B69] Cyberinfrastructure for Phylogenetic Research (CIPRES) (2021). The CIPRES Science Gateway.

[B70] National Center of Biotechnology Information (NCBI) (2022). Welcome to NCBI.

[B71] Orthoptera Species File (2022). Orthoptera Species File Online.

[B72] SimpleMappr https://www.simplemappr.net/.

